# Programmed Cell Death-1 Expression in T-Cell Subsets in Chickens Infected with Marek’s Disease Virus

**DOI:** 10.3390/pathogens14050431

**Published:** 2025-04-29

**Authors:** Jumpei Sato, Yoshinosuke Motai, Shunsuke Yamagami, Shwe Yee Win, Fumiya Horio, Hikaru Saeki, Naoya Maekawa, Tomohiro Okagawa, Satoru Konnai, Kazuhiko Ohashi, Shiro Murata

**Affiliations:** 1 Department of Disease Control, Faculty of Veterinary Medicine, Hokkaido University, Sapporo 060-0818, Japan; 2 Institute for Vaccine Research and Development, Hokkaido University, Sapporo 001-0021, Japan; 3 Veterinary Research Unit, International Institute for Zoonosis Control, Hokkaido University, Sapporo 001-0020, Japan; 4 International Affairs Office, Faculty of Veterinary Medicine, Hokkaido University, Sapporo 060-0818, Japan

**Keywords:** Marek’s disease virus, programmed cell death 1, Meq

## Abstract

Marek’s disease virus (MDV) causes Marek’s disease (MD) in chickens, characterized by malignant lymphomas and immunosuppression. Sporadic MD outbreaks continue to occur even among vaccinated flocks in certain regions due to the increased virulence of the field strains. However, the mechanisms of tumorigenesis and immunosuppression caused by MDV remain to be fully elucidated. We previously reported that the mRNA expression of programmed cell death 1 (PD-1), an immune checkpoint molecule, was increased in tumor lesions caused by MDV, and its expression was positively correlated with the mRNA expression of Meq, an MDV-specific oncogene. In this study, we characterized PD-1-expressing T-cell subsets in the spleen and tissues of chickens that developed tumors to investigate the association between PD-1 expression and immunosuppression. Flow cytometric analysis revealed that the proportion of PD-1-expressing CD4^+^ T-cells, which are targets of MDV tumorigenesis, increased in the spleen and tumor tissues of chickens with MD. The proportion of PD-1^+^ CD4^+^ T-cells was higher in Meq-expressing cells than in those that were not. In the spleens of chickens with MD, the proportions of PD-1-expressing cells were increased in CD8^+^ and γδ T-cells, which play pivotal roles in defense against MD pathogenesis, relative to those of spleens from uninfected chickens. Moreover, the proportion of PD-1^+^ CD8^+^ T-cells expressing interferon (IFN)-γ did not increase in the spleen of chickens with MD. Additionally, no difference in the proportion of IFN-γ^+^ γδ T-cells expressing and not expressing PD-1 was observed in the spleens of chickens with MD, although the proportion of IFN-γ^+^ γδ T-cells expressing PD-1 in the spleens of uninfected chickens was higher. The function of PD-1-expressing CD8^+^ and γδ T-cells in chickens may be impaired after developing MD, which may cause MDV-induced immunosuppression.

## 1. Introduction

Marek’s disease virus (MDV) is an avian alphaherpesvirus classified within the family *Orthoherpesviridae*, subfamily *Alphaherpesvirinae*, genus *Mardivirus*, and species *Mardivirus gallidalpha*2. It causes Marek’s disease (MD), including malignant lymphomas, neurological disorders, and immunosuppression. Although MD occurrences are currently prevented through vaccination with attenuated and/or non-pathogenic strains [1], the virulence of circulating field strains continues to increase. As a result, sporadic outbreaks of MD still occur, even in vaccinated flocks in certain regions [2,3,4,5,6,7]. Therefore, the development of vaccines with higher efficacy against MDV is urgently needed [8]. However, the mechanisms underlying tumorigenesis and immunosuppression induced by MDV remain incompletely understood.

The MDV life cycle comprises four phases [1]. Airborne cell-free MDV particles infect chickens through the respiratory tract along with dust and dander [2]. First, MDV infects macrophages residing in the lungs of infected chickens, and macrophages infected with it move to primary and secondary lymphoid tissues such as the thymus, bursa of Fabricius, and spleen [3,4,5]. The MDV causes semi-productive lytic replication of B, T, and NK cells in the primary lymphoid organs [6,7,8]. Following lytic replication, MDV establishes a latent infection in CD4^+^ T-cells, and only a few infected cells are subsequently transformed, resulting in lethal lymphoma development [9]. Most MDV-transformed tumor cells consist of CD4^+^ T-cells, indicating oligoclonal expansion of transformed CD4^+^ T-cells [2]. Meq, which is an MDV-specific oncogene that encodes a 339-amino-acid protein, is highly expressed in transformed CD4^+^ T-cells [10]. Meq is a transcription factor that forms a homodimer or heterodimer with AP-1 family proteins, such as c-Jun and Fos, through the leucine zipper [11]. It robustly upregulates genes involved in the v-Jun transforming pathway, such as JTAP-1, JAC, and HB-EGF [12], which are essential for the transformation of chicken CD4^+^ T-cells induced by MDV.

CD8^+^ T-cells play a pivotal role in cellular immunity, demonstrating effector functions that induce apoptosis in tumors and infected cells. Given that MDV is a highly cell-associated virus, CD8^+^ T-cells are thought to be crucial for the immune response against MDV. Supporting this, the depletion of CD8^+^ T-cells by administering monoclonal antibodies against chicken CD8 molecules resulted in an increased tumor incidence and reduced protective effect of vaccination in MDV-infected chickens [13]. Furthermore, the vaccine strain CVI988 induces memory CD8^+^ T-cells, which facilitate rapid proliferation and cytokine production upon subsequent MDV infection [14].

Interferon (IFN)-γ is a type II interferon that plays a key role in multiple protective functions against infections and tumor development, such as promotion of antigen processing and presentation, increased trafficking of leukocytes, induction of an antiviral state, and regulation of cellular proliferation and apoptosis [15,16,17]. A Th1 cell-driven inflammatory response mediated by IFN-γ is critical to prevent progression of the disease caused by the highly cell-associated MDV; indeed, some experiments have demonstrated that administering recombinant chicken IFN-γ to MDV-infected chickens delays disease progression and enhances vaccine-induced protection against MDV [18,19].

γδ T-cells comprise the majority of circulating and tissue-resident T-cells in chickens [20]. They can recognize both peptide and nonpeptide antigens upregulated in stressed cells in a major histocompatibility complex (MHC)-unrestricted manner and initiate rapid innate immune responses, unlike conventional αβ T-cells. In vivo experiments revealed a higher mortality rate of MD in γδ T-cell-depleted chickens, suggesting that γδ T-cells are involved in immunity against MDV infection [21]. Furthermore, CVI988 induces interferon (IFN)-γ^+^ γδ T-cells in the lung, spleen, and skin of chickens during the acute phase, and the adoptive transfer of PBMCs activated by chicken TCR γδ monoclonal antibody significantly decreases the tumor incidence among MDV-infected chickens [22,23]. In contrast, IFN-γ expression in γδ T-cells decreased in the supernatants from cultured splenocytes of MDV-challenged chickens [24]. Thus, immunosuppressive molecules, including interleukin (IL)-10 and transforming growth factor (TGF)-β, are released by MDV-transformed cells and may inhibit the potential effector functions of γδ T-cells within the tumor microenvironments of MDV-infected chickens. However, the mechanisms underlying the suppression of γδ T-cells in MDV-infected chickens remain unclear.

Programmed cell death 1 (PD-1) is an immune checkpoint molecule expressed on the surface of activated T and B cells [25]. PD-1 and its ligand, PD-Ligand 1 (PD-L1), have been identified in chickens and reported to suppress IFN-γ production from chicken T-cells [26]. The PD-1/PD-L1 pathway plays a critical role in immune evasion by tumor cells in human and animal cancers such as melanoma and leukemia [25,27,28,29]. Chronic viral infections also cause T-cell exhaustion. In infections with human T-cell leukemia virus and bovine leukemia virus, which are oncogenic retroviruses, both CD4^+^ and CD8^+^ T-cells progressively express PD-1 in accordance with disease progression, and exhausted T-cells fail to produce adequate cytokines [29,30]. Similarly, the PD-1/PD-L1 pathway is involved in immune evasion during MDV infection. In chickens infected with MDV, the level of expression of PD-1 mRNA increased in CD4^+^ and CD8^+^ T-cells during the early and secondary cytolytic phases [26,31]. Moreover, mRNA expressions of both PD-1 and PD-L1 were detected in tumor lesions of MDV-infected chickens by nested RT-PCR using laser-captured microdissections, and mRNA expression of PD-1 was positively correlated with mRNA expression of Meq [26], suggesting a higher expression of PD-1 in tumor cells. However, the expression of PD-1 and PD-L1 in tumor cells and other T-cell subsets infiltrating tumor lesions remains unknown, and the role of the PD-1/PD-L1 pathway in immunosuppression in MDV-infected chickens remains unclear.

We aimed to use flow cytometry to analyze the proportions of PD-1-expressing CD4^+^, CD8^+^, and γδ T-cells in the spleen and tumor tissues of chickens that developed lymphomas to investigate the association between PD-1 expression and immunosuppression induced by MDV. We also aimed to compare the proportions of PD-1-expressing cells in Meq-positive and negative CD4^+^ T-cells to assess PD-1 expression in MDV-infected cells of chickens that developed tumors. To investigate the effects of PD-1 expression on effector cells, we analyzed the proportion of IFN-γ-positive cells among PD-1-expressing CD8^+^ and γδ T-cells.

## 2. Materials and Methods

### 2.1. Ethics Statement

All animal experiments were approved by the Institutional Animal Care and Use Committee of Hokkaido University (approval number 22-0088). All experiments were performed in accordance with the relevant guidelines and regulations of the Faculty of Veterinary Medicine of Hokkaido University, which is fully accredited by the Association for Assessment and Accreditation of Laboratory Animal Care International.

### 2.2. Viruses Preparation

We used an RB-1B-based recombinant MDV (rMDV) as previously described [32,33]. The RB-1B genome was cloned as a bacterial artificial chromosome (BAC). The BAC used in this study encoded RB-1B-Meq in the terminal repeat long region and lacked the internal repeat long (IRL) region. The BAC was transfected into chicken embryo fibroblasts (CEFs) using a CalPhos Mammalian Transfection Kit (Takara Bio Inc., Kyoto, Japan) according to the manufacturer’s instructions. The pCAGGS-Cre plasmid was cotransfected to remove the BAC sequence from the viral genome. rMDV was passaged on CEFs and stored in Cell Banker 1 (Nippon Zenyaku Kogyo Co., Ltd., Fukushima, Japan) at −80 °C. As the IRL region is recovered in the process of virus reconstitution, its restoration, in addition to deleting the BAC sequence, was verified using PCR. The titer of reconstituted rMDV was determined using a plaque assay [34,35]. Finally, we sequenced the entire genome of the reconstituted recombinant viruses using next-generation sequencing (BioSample accession: LC849255) and confirmed that there were no mutations in the open reading frames that affect MDV virulence relative to the RB-1B parental strain.

### 2.3. Experimental Design

We analyzed the spleen and tumor tissues of chickens that developed MD during experimental infection. For the experimental infection, fertilized eggs (Iwamura Hatchery Co., Ltd., Niigata, Japan) from commercial chickens were hatched, and the chicks were raised in isolators at the animal facility of the Faculty of Veterinary Medicine of Hokkaido University. One-day-old chicks were inoculated via the intra-abdominal route with 5000 pfu of rMDV (*n* = 26) or phosphate-buffered saline (PBS) (pH 7.4) as a negative control (*n* = 10). After inoculating the chickens with rMDV, we monitored them daily for clinical signs of MD for 8 weeks. Moreover, attending veterinarians monitored the health status of chickens daily throughout the experimental period. This experimental infection was conducted by research staff who had received comprehensive training through an animal experimentation program administered by Hokkaido University. We set the humane endpoint at the onset of neurological symptoms such as leg paralysis, and chickens reaching this stage were euthanized on the same day. After deep general anesthesia with isoflurane (Zoetis Japan, Tokyo, Japan), all chickens were euthanized via cardiac blood collection, and heparinized blood samples were collected. Half of the chickens, including euthanized chickens on the last day of experimental infection, demonstrated solid tumor formation in visceral organs during the experimental period. The spleen and tumor tissues were collected from chickens that developed lymphoma between 28 and 56 days post-infection (dpi) and from the control group at 35 and 56 dpi for flow cytometric analysis. The spleen and tumor tissues formed in the kidneys or gonads were dissected with scissors, homogenized, and strained using 40-μm cell strainers (BD Biosciences, San Jose, CA, USA) to obtain cell suspensions. These were washed twice with PBS. Mononuclear cells were isolated from spleen cell suspensions via density gradient centrifugation using Percoll solution (GE Healthcare, Chicago, IL, USA) and washed twice with PBS. Isolated cells were stored in Cell Banker 1 (Nippon Zenyaku Kogyo Co., Ltd., Fukushima, Japan) for flow cytometric analysis.

### 2.4. Flow Cytometric Analysis

#### 2.4.1. Antibodies Against Meq and Chicken PD-1

For flow cytometric analysis, monoclonal antibodies (mAbs) against Meq were used (kindly provided by Dr. Kurokawa, National Agriculture and Food Research Organization, Tsukuba, Japan) [36]. Anti-Meq mAbs (mouse IgG1 isotype) were purified from the supernatant of cultured hybridomas using Protein G Sepharose 4 Fast Flow (GE Healthcare, Chicago, IL, USA) according to the protocol of the manufacturer. The purified antibody was conjugated with phycoerythrin (PE) using the Zenon Mouse IgG_1_ Labeling Kit (Invitrogen, Waltham MA, USA). To obtain the anti-chicken PD-1 polyclonal antibody, antisera were obtained from a rabbit immunized with a peptide corresponding to the 114–131 amino acid region of chicken PD-1 (accession number XP_422723) (Sigma-Aldrich, St. Louis, MI, USA).

#### 2.4.2. Expression of Meq and PD-1 in DF-1 Cells

The open reading frames of Meq derived from RB-1B (accession number: HM488349.1) and chicken PD-1 (accession number: XM422723) were amplified and cloned into a pCI-neo vector (Promega, Madison, WI, USA). The DF-1 cells were transfected with 300 ng of Meq or PD-1 expression plasmid using Lipofectamine 2000 (Thermo Fisher Scientific, Waltham, MA, USA) according to the manufacturer’s instructions. The availability of anti-Meq mAb or anti-PD-1 polyclonal antibodies for flow cytometric analysis was confirmed after 24 h by analyzing the expression of Meq and PD-1 in DF-1 cells transfected with the expression plasmids in the following manner using a FACSLyric flow cytometer (BD Biosciences).

#### 2.4.3. CD4^+^ T-Cell Staining

Mononuclear cells (5 × 10^5^) isolated from spleen and tumor tissues per well were seeded into 96-well plates and washed two times with PBS containing 1% bovine serum albumin (BSA; Sigma-Aldrich). To block nonspecific binding, cells were incubated with PBS containing 10% chicken serum (Thermo Fisher Scientific) at 25 °C for 15 min. Subsequently, they were stained with rabbit anti-chicken PD-1 polyclonal antibodies or negative rabbit serum for 30 min at 4 °C. After two washes with PBS containing 1% BSA, cells were stained with the following antibodies for 30 min at 4 °C in the dark: mouse anti-chicken CD3ε mAbs (PerCP-Cyanine5.5 (PerCP-Cy5.5), Southern Biotech Birmingham, AL, USA), mouse anti-chicken CD4 mAbs (PE-Cyanine7 dye (PE-Cy7); Southern Biotech), and mouse anti-rabbit IgG1 mAbs (allophycocyanin (APC); Thermo Fisher Scientific). Dead cells were removed by staining with Fixable Viability Dye eFluor780 (Thermo Fisher Scientific). After washing cells two times with PBS containing 1% BSA, they were fixed and permeabilized by resuspending with 200 μL of Cytofix/Cytoperm solution (BD Biosciences) for 30 min at 4 °C. After three washes with Perm/Wash buffer (BD Biosciences), cells were stained with mouse anti-Meq mAbs conjugated with PE or mouse IgG_1_ isotype mAbs (PE; Southern Biotech) for 40 min at 4 °C in the dark. After two final washes with Perm/Wash buffer, cells were resuspended in 300 μL of PBS containing 1% BSA and analyzed using a FACSLyric flow cytometer (BD Biosciences). The gating strategy is illustrated in Figure S1.

#### 2.4.4. CD8^+^ T-Cell and γδ T-Cell Staining

Mononuclear cells (5 × 10^5^) from spleen and tumor lesions per well were seeded into 96-well plates and cultured for 4 h at 41 °C and with 5% CO_2_ in RPMI-1640 medium (Sigma-Aldrich) containing brefeldin A (Sigma-Aldrich) to block protein transport and accumulate IFN-γ within the cells under unstimulated conditions. For stimulation, cells were incubated under the same conditions with RPMI-1640 medium containing brefeldin A, supplemented with phorbol 12-myristate 13-acetate (PMA; FUJIFILM Wako Pure Chemical Corporation, Osaka, Japan) and ionomycin (Sigma-Aldrich) as polyclonal stimulators to activate T-cells and induce IFN-γ production. After the incubation, cells were washed two times with PBS containing 1% BSA and blocked with PBS containing 10% chicken serum at 25 °C for 15 min. Unstimulated and stimulated cells were stained with rabbit anti-chicken PD-1 polyclonal antibodies or negative rabbit serum for 30 min at 4 °C. After washing two times with PBS containing 1% BSA, cells were stained with the following antibodies for 30 min at 4 °C in the dark: mouse anti-chicken CD3εmAbs (PerCP-Cy5.5; Southern Biotech), mouse anti-chicken γδ TCR mAbs (PE-Cy7; Southern Biotech), anti-chicken CD8β mAbs (fluorescein-5-isothiocyanate (FITC); Southern Biotech), and mouse anti-rabbit IgG1 mAb (APC; Thermo Fisher Scientific). Dead cells were removed by staining with Fixable Viability Dye eFluor780 (Thermo Fisher Scientific). For intracellular staining, after washing two times with PBS containing 1% BSA, cells were fixed and permeabilized by the treatment with Cytofix/Cytoperm solution (BD Biosciences) for 30 min at 4 °C, followed by three washes with Perm/Wash solution (BD Biosciences). Cells were then stained with mouse anti-chicken IFN-γ antibody conjugated with biotin (detection antibody from Chicken IFN-γ CytoSet™, Invitrogen) or mouse IgG_1_ isotype mAbs conjugated with biotin (BioLegend, San Diego, CA, USA) for 40 min at 4 °C in the dark, followed by two washes with Perm/Wash solution. Subsequently, they were stained with streptavidin (PE; Invitrogen) for 40 min at 4 °C in the dark and washed two times again. The cells were resuspended in 300 μL of PBS containing 1% BSA and analyzed using a FACSLyric flow cytometer (BD Biosciences). The gating strategy is illustrated in Figure S2.

### 2.5. Western Blotting

DF-1 cells were seeded in 24-well plates at a density of 2.0 × 10^5^ cells per well and incubated for 24 h. Cells were transfected with 300 ng of a PD-1 expression plasmid using Lipofectamine 2000 (Thermo Fisher Scientific) according to the manufacturer’s instructions. Twenty-four hours post-transfection, the cells were harvested and lysed using Laemmli sample buffer (Bio-Rad, Hercules, CA, USA) supplemented with 5% 2-mercaptoethanol (Sigma-Aldrich). The lysates were denatured by heating at 99 °C for 5 min and subsequently separated using 10% sodium dodecyl sulfate-polyacrylamide gel electrophoresis. Subsequently, proteins were transferred onto a nitrocellulose membrane (Sigma-Aldrich). The membrane was blocked with phosphate-buffered saline containing Tween 20 (PBS-T) and 3% skim milk for 1 h at room temperature. It was then incubated for 1 h with rabbit anti-chicken PD-1 antisera (20 μg/mL) and mouse anti-chicken actin antibody (20 μg/mL) as an internal control. After three washes with PBS-T, the membrane was incubated with either an HRP-conjugated anti-rabbit IgG secondary antibody (Promega) or an HRP-conjugated anti-mouse IgG1 secondary antibody (Thermo Fisher Scientific) for 30 min at room temperature. Following additional washes with PBS-T, protein bands were visualized using a chemiluminescent detection system for Western blotting.

### 2.6. Statistical Analyses

Statistical analyses were conducted using the R Statistical Software (version 4.0.3; R Foundation for Statistical Computing, Vienna, Austria). Differences in the proportion of T-cell subsets in chickens were analyzed using Dunn’s test. The proportions of IFN-γ ^+^ cells within the same individual were compared using the Wilcoxon signed-rank sum test. Statistical significance was set at *p* < 0.05.

## 3. Results

### 3.1. CD4^+^ T-Cells Transformed by MDV Express a High Proportion of PD-1

**Figure 1:**
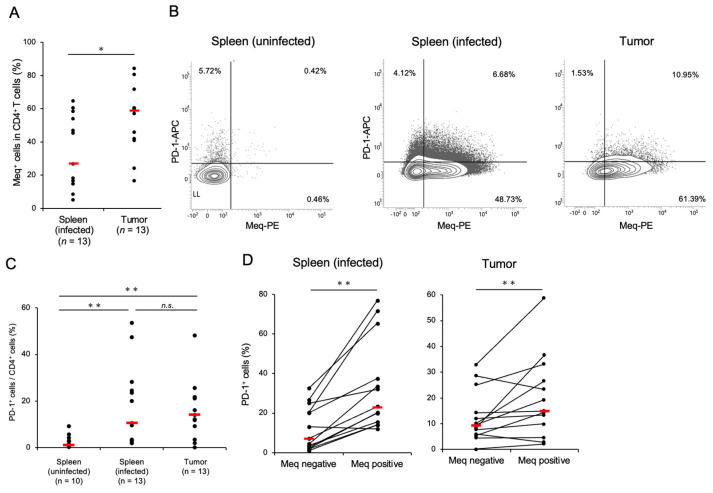
CD4^+^ T-cells transformed by Marek’s disease virus express a high proportion of programmed cell death 1. The expression of PD-1 and Meq in CD4^+^ T-cells was analyzed (control group: *n* = 10, spleen of MDV-infected chicken: *n* = 13, tumor tissue of MDV-infected chicken: *n* = 13). (**A**) The proportions of Meq^+^ cells in CD4^+^ T-cells in the spleen and tumor tissues were analyzed. (**B**) Representative flow cytometry plots showing PD-1 and Meq expressions in CD4^+^ T-cells. (**C**) The proportions of PD-1^+^ cells in the total CD4^+^ cells in the spleen and tumor tissues were compared. (**D**) The proportions of PD-1^+^ cells between Meq^+^ CD4^+^ and Meq^−^ CD4^+^ cells in the spleens of uninfected and infected chickens were compared. The red line indicates the mean value. Asterisks indicate significant differences (** *p* < 0.01, * *p* < 0.05; Dunn’s test between the groups; Wilcoxon signed-rank sum within the same individual).

Chickens were infected with rMDV to assess PD-1 expression in T-cell subsets in those that developed tumors. Thirteen of 26 infected chickens developed tumors in visceral organs. The spleen and tumor tissues in chickens with MD showed a higher proportion of CD4^+^ T-cells than those of the uninfected controls (Figure S3A). Kurokawa et al. previously established mAbs against Meq for histopathological examination [36,37], and we confirmed the availability of this mAb by intracellular staining using DF-1 cells transfected with the Meq expression plasmid (Figure S4A). We applied the anti-Meq mAb for the analysis of Meq^+^ cells in the spleen and tumor tissues of chickens that had developed MD. The Meq^+^ CD4^+^ T-cells were detected in both the spleen and tumor tissues of infected chickens, and the proportion of Meq^+^ CD4^+^ T-cells was significantly higher in tumor tissues than in the spleens (Figure 1A). These data suggest that both the spleen and tumor tissues of chickens developing MD had MDV-transformed CD4^+^ T-cells.

To investigate PD-1 expression in CD4^+^ T-cells of MDV-infected chickens, we generated anti-chicken PD-1 polyclonal antibodies and successfully detected PD-1 expression in DF-1 cells transfected with the PD-1 expression plasmid using flow cytometry and western blotting (Figure S4B,C). To analyze PD-1 expression in MDV-transformed CD4^+^ T-cells, we compared the proportions of PD-1^+^ cells in the total CD4^+^ T-cells in the spleen and tumor tissues of chickens with MD. Compared to uninfected chickens, the proportion of PD-1^+^ CD4^+^ T-cells was significantly higher in the spleen and tumor tissues of chickens with MD than in those without MD (Figure 1B,C). When we compared the proportion of PD-1^+^ cells between Meq^+^ cells and Meq^−^ cells, the proportion of PD-1^+^ cells was significantly higher in Meq^+^ CD4^+^ T-cells than in Meq^−^ CD4^+^ T-cells in the spleen and tumor tissues of chickens with MD (Figure 1B,D). Thus, the proportion of PD-1^+^ cells, especially in transformed CD4^+^ T-cells expressing Meq, increased in the spleen and tumor tissues of chickens with MD.

### 3.2. Expressions of IFN-γ and PD-1 in CD8^+^ T-Cells in Chickens with MD

**Figure 2:**
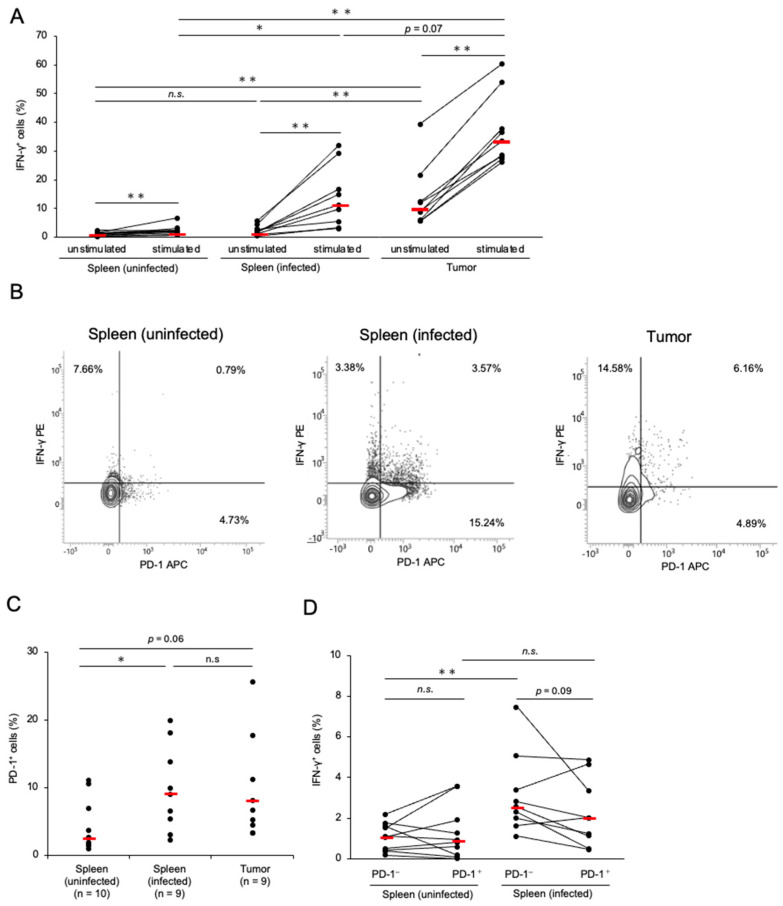
Expressions of interferon-γ and programmed cell death 1 in CD8^+^ T-cells in Marek’s disease-developed chickens. The expressions of PD-1 and IFN-γ in CD8^+^ T-cells were analyzed (control group: *n* = 10, spleen of MDV-infected chicken: *n* = 9, tumor tissue of MDV-infected chicken: *n* = 9). (**A**) The proportions of IFN-γ^+^ cells in the total CD8^+^ T-cells from the spleen and tumor tissues were determined after unstimulated and stimulated culture. (**B**) Representative flow cytometry plots showing the upregulation of PD-1 and IFN-γ in CD8^+^ T-cells. (**C**) The proportion of PD-1^+^ cells in the CD8^+^ T-cell population from the spleen and tumor tissues was analyzed. (**D**) The proportions of IFN-γ^+^ cells between PD-1^+^ CD8^+^ and PD-1^−^ CD8^+^ T-cells in the spleens of uninfected and infected chickens were compared. The red line indicates the mean value. Asterisks indicate significant differences (** *p* < 0.01, * *p* < 0.05; Dunn’s test between the groups; Wilcoxon signed-rank sum within the same individual).

We determined the proportion of CD8^+^ T-cells expressing IFN-γ to investigate the cytotoxic potential of CD8^+^ T-cells in chickens with MD. Compared with the control group, the proportion of CD8^+^ T-cells was decreased in the spleen and tumor tissues of the chickens with MD (Figure S3B). This was presumed to be due to the increase in the number of transformed CD4^+^ T-cells. The percentage of IFN-γ^+^ CD8^+^ T-cells in tumor tissues was significantly higher than that in the spleens of the control and infected chickens (Figure 2A). In contrast, the proportion of IFN-γ^+^ CD8^+^ T-cells in the spleens of infected chickens was comparable to that in the control group (Figure 2A). To assess whether CD8^+^ T-cells in chickens with MD still produce IFN-γ, we stimulated isolated cells with PMA and ionomycin and analyzed the proportion of IFN-γ^+^ CD8^+^ T-cells. The proportion of IFN-γ^+^ CD8^+^ T-cells in stimulated cells was increased relative to that in unstimulated cells in all groups. Therefore, the CD8^+^ T-cells in the chickens with MD seemed to possess the function of IFN-γ production in response to mitogen stimulation. Notably, the proportion of IFN-γ^+^ CD8^+^ T-cells in the spleen of chickens with MD was significantly higher than that in the control group after stimulation, although no significant difference was observed between them under unstimulated conditions.

Next, we analyzed PD-1 expression in the CD8^+^ T-cells of chickens with MD. The proportion of PD-1^+^ CD8^+^ T-cells in the spleens of infected chickens was significantly higher than that in the control group, and the proportion of PD-1^+^ CD8^+^ T-cells in the tumor tissues tended to be higher than that in the control group (*p* = 0.062, Figure 2B,C). To assess the cytotoxic potential of PD-1^+^ CD8^+^ T-cells in chickens with MD, we compared the proportion of IFN-γ^+^ cells in PD-1^+^ CD8^+^ T-cells with that in PD-1^−^ CD8^+^ T-cells in the spleens of uninfected and infected chickens. The proportion of IFN-γ^+^ cells in PD-1^−^ CD8^+^ T-cells in the spleens of chickens with MD was increased relative to that in the uninfected chickens; however, no difference was observed between the proportions of IFN-γ^+^ cells in PD-1^+^ CD8^+^ T-cells in the spleens of uninfected and infected chickens (Figure 2B,D). These findings suggest that the cytokine production of PD-1^+^ CD8^+^ T-cells is impaired in chickens with MD.

### 3.3. Expressions of IFN-γ and PD-1 in γδ T-Cells in Chickens with MD

**Figure 3:**
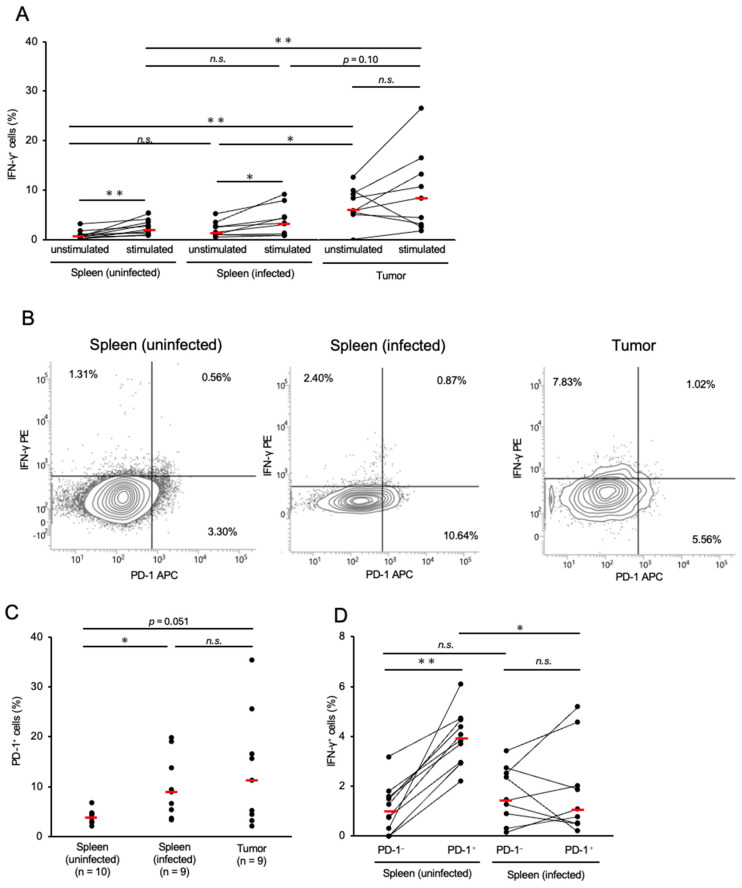
Expressions of interferon-γ and programmed cell death 1 in γδ T-cells in Marek’s disease-developed chickens. The expressions of PD-1 and IFN-γ in γδ T-cells were analyzed (control group: *n* = 10, spleen of MD virus (MDV)-infected chicken: *n* = 9, tumor tissue of MDV-infected chicken: *n* = 9). (**A**) The proportions of γδ T-cells expressing IFN-γ in the spleen and tumor tissues were determined after unstimulated and stimulated culture. (**B**) Representative flow cytometry plots showing the upregulation of PD-1 and IFN-γ in γδ T-cells. (**C**) The proportion of PD-1^+^ γδ T-cells in the spleen and tumor tissues was determined. (**D**) The proportions of IFN-γ^+^ cells between PD-1^+^ and PD-1^−^ γδ T-cells in the spleens of uninfected and infected chickens were compared. The red line indicates the mean value. Asterisks indicate significant differences (** *p* < 0.01, * *p* < 0.05; Dunn’s test between the groups; Wilcoxon signed-rank sum within the same individual).

The proportions of γδ T-cells in the spleen and tumor tissues of infected chickens were significantly lower than those in the control group (Figure S3C), which were similar to that of CD8^+^ T-cells. The proportion of IFN-γ^+^ γδ T-cells in the tumor tissues was significantly higher than that in the spleens of the control and infected groups (Figure 3A). Conversely, the proportion of IFN-γ^+^ γδ T-cells in the spleens of the infected chickens was comparable to that in the control group (Figure 3A). We further assessed IFN-γ production by γδ T-cells in response to mitogen stimulation. In the spleens of both the control and MDV-infected groups, the proportion of IFN-γ^+^ γδ T-cells significantly increased following stimulation (Figure 3A). However, no significant difference was observed between the proportions of IFN-γ^+^ γδ T-cells in the stimulated and unstimulated cultures (Figure 3A).

Similar to conventional αβ T-cells, γδ T-cells can become exhausted following chronic exposure to the antigens of pathogens or tumors [38]. Therefore, we analyzed the proportion of PD-1^+^ cells among the total γδ T-cells in chickens with MD. The spleens of infected chickens had significantly higher proportions of PD-1^+^ γδ T-cells than the spleens of controls (Figure 3B,C). The proportion of PD-1^+^ γδ T-cells in the tumor tissues was also higher than that in the control group (*p* = 0.051, Figure 3C). To investigate the relationship between PD-1 and IFN-γ expression in γδ T-cells, we compared the proportions of IFN-γ^+^ cells in PD-1^+^ and PD-1^−^ γδ T-cells in the spleens of uninfected and infected chickens. In the spleens of the control group, PD-1^+^ γδ T-cells showed a significantly higher proportion of IFN-γ^+^ cells than PD-1^−^ γδ T-cells. In contrast, no significant difference in the proportion of IFN-γ^+^ cells between PD-1^+^ and PD-1^−^ γδ T-cells was observed in the spleens of the MD group. However, the proportion of IFN-γ^+^ cells in PD-1^+^ γδ T-cells was significantly lower in chickens with MD than in the control group (Figure 3B,D). Collectively, PD-1^+^ γδ T-cells in chickens with MD may have impaired cytotoxicity.

## 4. Discussion

In this study, we observed an increased proportion of PD-1-expressing CD4^+^ T-cells in MDV-infected chickens, and Meq^+^ CD4^+^ T-cells had significantly higher PD-1 expression than Meq^−^ CD4^+^ T-cells. Additionally, a higher proportion of CD8^+^ T-cells and γδ T-cells infiltrating the tumor tissues expressed IFN-γ than those in the spleens of uninfected and infected chickens. In CD8^+^ cells, the proportion of IFN-γ^+^ cells in PD1^−^ cells was higher in the spleens of infected chickens relative to those in uninfected chickens, whereas no difference was observed in the proportion of IFN-γ^+^ cells in PD1^+^ cells in the spleens between uninfected and infected chickens. The proportion of IFN-γ^+^ γδ T-cells in the tumor tissues did not increase in response to mitogen stimulation. Moreover, no difference in the proportion of IFN-γ^+^ γδ T-cells in the spleens of chickens with MD was observed, regardless of PD-1 expression, whereas the proportion of IFN-γ^+^ γδ T-cells was higher in PD-1^+^ cells in the spleens of uninfected chickens. These observations suggest that tumor cells have a PD-1-expressing phenotype, whereas the immune response against the development of MD appears to be diminished in PD-1-expressing CD8^+^ and γδ T-cells.

In the present study, the proportion of PD-1-expressing cells was significantly higher in Meq^+^ CD4^+^ T-cells than in Meq^−^ CD4^+^ T-cells, suggesting that MDV-transformed CD4^+^ T-cells highly express PD-1. Two hypotheses may explain this observation. First, MDV may selectively transform CD4^+^ T-cells expressing PD-1. In humans and mice, activated T-cells express high levels of PD-1 [39], and MDV-transformed cells express CD25, another marker of T-cell activation [40]. Therefore, MDV may preferentially establish latent infection in activated CD4^+^ T-cells expressing PD-1 and transform them. Second, MDV-transformed cells may express PD-1 in response to the inducer of PD-1 expression, which is secreted by immune cells in the tumor microenvironment. PD-1 expression is regulated by IFN-γ in both humans and mice [41]. We previously reported that PD-1 mRNA expression was correlated with IFN-γ mRNA expression in tumor lesions of MDV-infected chickens [26]. Additionally, the proportion of IFN-γ-expressing CD8^+^ T-cells and γδ T-cells was higher in the tumor tissues than in the spleens of uninfected and infected chickens in this study. Thus, tumor cells may express PD-1 in response to inducers such as IFN-γ from CD8^+^ T-cells and γδ T-cells in the tumor microenvironment. Thus, PD-1 expression in MDV-transformed CD4^+^ T-cells may reflect cellular characteristics before transformation and the factors in the tumor microenvironment. PD-1 mRNA expression levels in MDV-transformed cell lines, including MDCC-MSB1 and HP1, have been reported to be lower than those in PBMCs from uninfected chickens [26]. This observation may support the hypothesis that PD-1 expression is induced in response to factors present in the tumor microenvironment. Alternatively, PD-1 expression can decline over time without antigen stimulation [42], suggesting that MDCC-MSB1 cells lose expression during long-term culture. On the other hand, PD-1 expression may be beneficial for tumor cells. MDV-transformed T-cells have a phenotype resembling regulatory T-cells (Treg cells) expressing a membrane-bound form of TGF-β [43,44]. In mice with chronic lymphocytic choriomeningitis virus infection, PD-1 expression is increased in Treg cells, and the interaction between PD-1 on Treg cells and PD-L1 on CD8^+^ T-cells suppresses the proliferation of CD8^+^ T-cells and subsequent cytokine production [45]. Therefore, PD-1 expressed on MDV-transformed CD4^+^ T-cells might be involved in the suppression of cytokine production by CD8^+^ T-cells. Further research is necessary to determine the function of PD-1 expressed on transformed T-cells.

No significant difference between the proportions of IFN-γ-expressing CD8^+^ T-cells in the spleens of uninfected and infected chickens was observed under unstimulated conditions. However, the proportion of IFN-γ^+^ CD8^+^ T-cells was elevated in the spleens of infected chickens following stimulation with mitogen for 4 h, and was significantly higher than that in uninfected chickens. According to previous reports, transformed cells downregulate MHC class I [6], thereby contributing to evasion from effector T-cells. Additionally, the responsiveness of CD8^+^ T-cells to stimulation through T-cell receptors (TCR) is decreased in MDV-infected chickens [6]. The PMA and ionomycin used for stimulation in this study bypass the TCR signaling pathway and directly activate NF-κB in T-cells. This suggested that CD8^+^ T-cells, which had a diminished response to stimulation from the TCR, may have produced IFN-γ after stimulation with PMA and ionomycin. Thus, some of the CD8^+^ T-cells in the spleens of chickens with MD may represent diminished functions, such as the recognition of tumor cells and cytokine production.

In agreement with previous studies demonstrating increased expression of PD-1 mRNA in CD8^+^ T-cells in MDV-infected chickens [31], our flow cytometric analysis revealed an increased proportion of PD-1-expressing CD8^+^ T-cells in the spleen and tumor tissues of chickens with MD. In the chickens with MD, the proportion of IFN-γ-expressing cells in PD-1^−^ CD8^+^ T-cells in the spleen was higher than that in uninfected chickens, suggesting an increase in the population of CD8^+^ T-cells specific to infected and/or tumor cells. However, no difference in the proportion of IFN-γ-expressing cells in PD-1^+^ CD8^+^ T-cells in the spleens of uninfected and infected chickens was observed. This suggested that IFN-γ expression by CD8^+^ T-cells was reduced during PD-1 expression in chickens with MD. In the spleen and tumor tissues of chickens with MD, transformed and infected cells are abundant. Chronic exposure to viral and tumor antigens expressed by these cells may drive T-cell exhaustion, particularly in CD8⁺ and γδ T-cells. This phenomenon, commonly observed in chronic viral infections and cancers, is characterized by the upregulation of PD-1 and a loss of T-cell effector functions. In this study, we observed a decreased IFN-γ production on PD-1^+^ CD8⁺ T-cells in chickens with advanced disease, compared to control chickens, suggesting that chronic stimulation induces functional exhaustion of CD8^+^ T-cells. The impaired cytokine production on T-cells could contribute to the immunosuppression observed in recent highly virulent field strains. Further investigation, including the response of CD8^+^ T-cells to viral antigens, such as cytokine production and PD-1 expression on antigen-specific CD8^+^ T-cells, is required to characterize the reduced function and exhaustion of CD8^+^ T-cells.

Previous studies have demonstrated that supernatants from cultured splenocytes of MDV-infected chickens reduce IFN-γ expression in γδ T-cells [24]. In the present study, we observed that the proportion of IFN-γ^+^ γδ T-cells in tumor tissues did not increase in response to mitogen stimulation, whereas the proportion of IFN-γ^+^ γδ T-cells in the spleens of infected and uninfected chickens was increased by stimulation. These data suggest that the functions of γδ T-cells may be more strongly impaired by cells comprising the tumor microenvironment. In the spleens of uninfected chickens, PD-1^+^ γδ T-cells showed a significantly higher proportion of IFN-γ-expressing cells than PD-1^−^ γδ T-cells, and some of the γδ T-cells expressing PD-1 are present in uninfected chickens. These cells seem to exhibit an IFN-γ-producing phenotype. In contrast, in the spleens of chickens with MD, no increase in the proportion of IFN-γ-expressing cells in PD-1^+^ γδ T-cells was observed relative to that in PD-1^−^ γδ T-cells. These results also suggest that the function of PD-1^+^ γδ T-cells is impaired in chickens with MD. Exhausted γδ T-cells in humans and mice express other immune checkpoint molecules, such as cytotoxic T-lymphocyte-associated protein 4 (CTLA-4) and T-cell immunoglobulin and mucin-domain containing-3 (Tim-3), in addition to PD-1 [46,47]. Further analysis, including the expression of other immune checkpoint molecules on γδ T-cells, is required to better understand the functions of γδ T-cells in MD pathogenesis.

In this study, we analyzed PD-1 expression in T-cell subsets from experimentally infected chickens. We previously reported that the expression of PD-1 mRNA was increased in samples from chickens that developed MD on commercial farms [26]. Unlike animal experiments, which reproduce pathological conditions, a longer period is expected until the development of MD in the field. Therefore, in chickens that developed MD in the field, CD8^+^ and γδ T-cells may be exposed to viral and tumor antigens for longer periods and express PD-1, potentially leading to more advanced exhaustion. In the present study, we did not compare the proportions of IFN-γ-expressing cells between PD-1^+^ cells and PD-1^−^ cells in tumor tissues because of the insufficient CD8^+^ and γδ T-cells. CD8^+^ and γδ T-cells in tumor tissues showed a higher proportion of IFN-γ-expressing cells than those in the spleens of infected chickens; therefore, more antigen-specific CD8^+^ and γδ T-cells are thought to infiltrate the tumor microenvironment and have more opportunities to be exposed to viral and tumor antigens. Further studies, such as single-cell RNA sequencing, are necessary to elucidate the involvement of PD-1 in immunosuppression in the tumor microenvironment.

Although spleens from uninfected chickens were used as negative controls in this study, we acknowledge that they are not a perfect negative control for tumor tissues due to differences in tissue structure and microenvironment. However, the spleen is a secondary lymphoid organ where lymphocytes actively respond to pathogens and produce cytokines such as IFN-γ. Thus, they provide an immunological baseline for evaluating changes in T-cell subsets in chickens with MD. Among the available tissues in chickens, we consider the spleen to be the most suitable control tissue for comparing IFN-γ production and PD-1 expression in each T-cell subset within tumor tissues.

In this study, we confirmed an increased proportion of PD-1-expressing CD4^+^, CD8^+^, and γδ T-cells in chickens with MD. Furthermore, our data on IFN-γ-expressing PD-1^+^ CD8^+^ T-cells and PD-1^+^ γδ T-cells suggest a relationship between PD-1 expression and diminished function of these T-cell subsets. This study provides insights into the pathogenesis of MDV and contributes to our understanding of the immune response to MDV in chickens. To further characterize the roles of the PD-1/PD-L1 pathway in MD pathogenesis, it is desirable to establish antibodies against PD-1 ligands such as PD-L1 and analyze their expression using these antibodies.

## Data Availability

The original contributions presented in this study are included in the article/Supplementary Materials. Further inquiries can be directed to the corresponding author.

## Supplementary Materials

The following supporting information can be downloaded at: https://www.mdpi.com/article/10.3390/pathogens14050431/s1, Figure S1: Gating strategy for analyzing CD4^+^ T-cells; Figure S2: Gating strategy for analyzing CD8^+^ T-cells and γδ T-cells; Figure S3: The percentages of T-cell subsets in the spleen and tumor tissue; Figure S4: Verification of binding affinity of programmed cell death-1 and Meq antibodies.

## References

[1] Calnek B.W., Hirai K. (2001). Pathogenesis of Marek’s Disease Virus Infection. Marek’s Disease.

[2] Abdul-Careem M.F., Javaheri-Vayeghan A., Shanmuganathan S., Haghighi H.R., Read L.R., Haq K., Hunter D.B., Schat K.A., Heidari M., Sharif S. (2009). Establishment of an aerosol-based Marek’s disease virus infection model. Avian Dis..

[3] Barrow A.D., Burgess S.C., Baigent S.J., Howes K., Nair V.K. (2003). Infection of macrophages by a lymphotropic herpesvirus: A new tropism for Marek’s disease virus. J. Gen. Virol..

[4] Davison F., Nair V. (2005). Use of Marek’s disease vaccines: Could they be driving the virus to increasing virulence?. Expert Rev. Vaccines.

[5] Baaten B.J., Staines K.A., Smith L.P., Skinner H., Davison T.F., Butter C. (2009). Early replication in pulmonary B cells after infection with Marek’s disease herpesvirus by the respiratory route. Viral Immunol..

[6] Morimura T., Ohashi K., Kon Y., Hattori M., Sugimoto C., Onuma M. (1996). Apoptosis and CD8-down-regulation in the thymus of chickens infected with Marek’s disease virus. Arch. Virol..

[7] Berthault C., Larcher T., Härtle S., Vautherot J.-F., Trapp-Fragnet L., Denesvre C. (2018). Atrophy of primary lymphoid organs induced by Marek’s disease virus during early infection is associated with increased apoptosis, inhibition of cell proliferation and a severe B-lymphopenia. Vet. Res..

[8] Bertzbach L.D., van Haarlem D.A., Härtle S., Kaufer B.B., Jansen C.A. (2019). Marek’s Disease Virus Infection of Natural Killer Cells. Microorganisms.

[9] Mwangi W.N., Smith L.P., Baigent S.J., Beal R.K., Nair V., Smith A.L. (2011). Clonal Structure of Rapid-Onset MDV-Driven CD4+ Lymphomas and Responding CD8+ T Cells. Osterrieder N, editor. PLoS Pathog..

[10] Liu J.L., Kung H.J. (2000). Marek’s disease herpesvirus transforming protein MEQ: A c-Jun analogue with an alternative life style. Virus Genes.

[11] Qian Z., Brunovskis P., Lee L., Vogt P.K., Kung H.J. (1996). Novel DNA binding specificities of a putative herpesvirus bZIP oncoprotein. J. Virol..

[12] Levy A.M., Gilad O., Xia L., Izumiya Y., Choi J., Tsalenko A., Yakhini Z., Witter R., Lee L., Cardona C.J. (2005). Marek’s disease virus Meq transforms chicken cells via the v-Jun transcriptional cascade: A converging transforming pathway for avian oncoviruses. Proc. Natl. Acad. Sci. USA.

[13] Umthong S., Dunn J.R., Cheng H.H. (2020). Depletion of CD8αβ+ T Cells in Chickens Demonstrates Their Involvement in Protective Immunity towards Marek’s Disease with Respect to Tumor Incidence and Vaccinal Protection. Vaccines.

[14] Hao X., Li S., Li J., Yang Y., Qin A., Shang S. (2021). An Anti-Tumor Vaccine Against Marek’s Disease Virus Induces Differential Activation and Memory Response of γδ T Cells and CD8 T Cells in Chickens. Front. Immunol..

[15] Kak G., Raza M., Tiwari B.K. (2018). Interferon-gamma (IFN-γ): Exploring its implications in infectious diseases. Biomol. Concepts.

[16] Sadler A.J., Williams B.R. (2008). Interferon-inducible antiviral effectors. Nat. Rev. Immunol..

[17] Jorgovanovic D., Song M., Wang L., Zhang Y. (2020). Roles of IFN-γ in tumor progression and regression: A review. Biomark. Res..

[18] Bertzbach L.D., Harlin O., Härtle S., Fehler F., Vychodil T., Kaufer B.B., Kaspers B. (2019). IFNα and IFNγ Impede Marek’s Disease Progression. Viruses.

[19] Haq K., Elawadli I., Parvizi P., Mallick A.I., Behboudi S., Sharif S. (2011). Interferon-γ influences immunity elicited by vaccines against very virulent Marek’s disease virus. Antivir. Res..

[20] Meijerink N., van Haarlem D.A., Velkers F.C., Stegeman A.J., Rutten V.P.M.G., Jansen C.A. (2021). Analysis of chicken intestinal natural killer cells, a major IEL subset during embryonic and early life. Dev. Comp. Immunol..

[21] Sabsabi M.A., Kheimar A., You Y., von La Roche D., Härtle S., Göbel T.W., von Heyl T., Schusser B., Kaufer B.B. (2024). Unraveling the role of γδ T cells in the pathogenesis of an oncogenic avian herpesvirus. mBio.

[22] Matsuyama-Kato A., Iseki H., Boodhoo N., Bavananthasivam J., Alqazlan N., Abdul-Careem M.F., Plattner B.L., Behboudi S., Sharif S. (2022). Phenotypic characterization of gamma delta (γδ) T cells in chickens infected with or vaccinated against Marek’s disease virus. Virology.

[23] Matsuyama-Kato A., Shojadoost B., Boodhoo N., Raj S., Alizadeh M., Fazel F., Fletcher C., Zheng J., Gupta B., Abdul-Careem M.F. (2023). Activated Chicken Gamma Delta T Cells Are Involved in Protective Immunity against Marek’s Disease. Viruses.

[24] Matsuyama-Kato A., Boodhoo N., Raj S., Abdul-Careem M.F., Plattner B.L., Behboudi S., Sharif S. (2023). The tumor microenvironment generated by Marek’s disease virus suppresses interferon-gamma-producing gamma delta T cells. Vet. Microbiol..

[25] Freeman G.J., Long A.J., Iwai Y., Bourque K., Chernova T., Nishimura H., Fitz L.J., Malenkovich N., Okazaki T., Byrne M.C. (2000). Engagement of the PD-1 immunoinhibitory receptor by a novel B7 family member leads to negative regulation of lymphocyte activation. J. Exp. Med..

[26] Matsuyama-Kato A., Murata S., Isezaki M., Kano R., Takasaki S., Ichii O., Konnai S., Ohashi K. (2012). Molecular characterization of immunoinhibitory factors PD-1/PD-L1 in chickens infected with Marek’s disease virus. Virol. J..

[27] Kleffel S., Posch C., Barthel S.R., Mueller H., Schlapbach C., Guenova E., Elco C.P., Lee N., Juneja V.R., Zhan Q. (2015). Melanoma Cell-Intrinsic PD-1 Receptor Functions Promote Tumor Growth. Cell.

[28] Maekawa N., Konnai S., Okagawa T., Nishimori A., Ikebuchi R., Izumi Y., Takagi S., Kagawa Y., Nakajima C., Suzuki Y. (2016). Immunohistochemical Analysis of PD-L1 Expression in Canine Malignant Cancers and PD-1 Expression on Lymphocytes in Canine Oral Melanoma. PLoS ONE.

[29] Ikebuchi R., Konnai S., Sunden Y., Onuma M., Ohashi K. (2010). Molecular cloning and expression analysis of bovine programmed death-1. Microbiol. Immunol..

[30] Kozako T., Yoshimitsu M., Fujiwara H., Masamoto I., Horai S., White Y., Akimoto M., Suzuki S., Matsushita K., Uozumi K. (2009). PD-1/PD-L1 expression in human T-cell leukemia virus type 1 carriers and adult T-cell leukemia/lymphoma patients. Leukemia.

[31] Parvizi P., Andrzejewski K., Read L.R., Behboudi S., Sharif S. (2010). Expression profiling of genes associated with regulatory functions of T-cell subsets in Marek’s disease virus-infected chickens. Avian Pathol..

[32] Sato J., Murata S., Yang Z., Kaufer B.B., Fujisawa S., Seo H., Maekawa N., Okagawa T., Konnai S., Osterrieder N. (2022). Effect of Insertion and Deletion in the Meq Protein Encoded by Highly Oncogenic Marek’s Disease Virus on Transactivation Activity and Virulence. Viruses.

[33] Conradie A.M., Bertzbach L.D., Bhandari N., Parcells M., Kaufer B.B. (2019). A Common Live-Attenuated Avian Herpesvirus Vaccine Expresses a Very Potent Oncogene. Goodrum F, editor. mSphere.

[34] Schumacher D., Tischer B.K., Fuchs W., Osterrieder N. (2000). Reconstitution of Marek’s Disease Virus Serotype 1 (MDV-1) from DNA Cloned as a Bacterial Artificial Chromosome and Characterization of a Glycoprotein B-Negative MDV-1 Mutant. J. Virol..

[35] Jarosinski K.W., Schat K.A. (2007). Multiple alternative splicing to exons II and III of viral interleukin-8 (vIL-8) in the Marek’s disease virus genome: The importance of vIL-8 exon I. Virus Genes.

[36] Kurokawa A., Yamamoto Y. (2022). Development of monoclonal antibodies specific to Marek disease virus-EcoRI-Q (Meq) for the immunohistochemical diagnosis of Marek disease using formalin-fixed, paraffin-embedded samples. J. Vet. Diagn. Investig..

[37] Kurokawa A., Yamamoto Y. (2024). Immunohistochemical Diagnosis of Marek’s Disease Based on Meq Detection in 104 Field Cases of Chicken Lymphoma. Avian Dis..

[38] Chen D., Guo Y., Jiang J., Wu P., Zhang T., Wei Q., Huang J., Wu D. (2022). γδ T cell exhaustion: Opportunities for intervention. J. Leukoc. Biol..

[39] Raimondi G., Shufesky W.J., Tokita D., Morelli A.E., Thomson A.W. (2006). Regulated compartmentalization of programmed cell death-1 discriminates CD4+CD25+ resting regulatory T cells from activated T cells. J. Immunol..

[40] Burgess S.C., Davison T.F. (2002). Identification of the Neoplastically Transformed Cells in Marek’s Disease Herpesvirus-Induced Lymphomas: Recognition by the Monoclonal Antibody AV37. J. Virol..

[41] Keir M.E., Butte M.J., Freeman G.J., Sharpe A.H. (2008). PD-1 and Its Ligands in Tolerance and Immunity. Annu. Rev. Immunol..

[42] Sheng M.K., Vick L., Collins C., Yoon D.J., Murphy W.J. (2023). Asymmetrical Expression of PD1 and CD25 in T-Cells Post-Initial Activation. J. Immunol..

[43] Gurung A., Kamble N., Kaufer B.B., Pathan A., Behboudi S. (2017). Association of Marek’s Disease induced immunosuppression with activation of a novel regulatory T cells in chickens. Cheng HH, editor. PLoS Pathog..

[44] Buza J.J., Burgess S.C. (2007). Modeling the proteome of a Marek’s disease transformed cell line: A natural animal model for CD30 overexpressing lymphomas. Proteomics.

[45] Park H.J., Park J.S., Jeong Y.H., Son J., Ban Y.H., Lee B.H., Chen L., Chang J., Chung D.H., Choi I. (2015). PD-1 upregulated on regulatory T cells during chronic virus infection enhances the suppression of CD8+ T cell immune response via the interaction with PD-L1 expressed on CD8+ T cells. J. Immunol..

[46] Brauneck F., Weimer P., Schulze zur Wiesch J., Weisel K., Leypoldt L., Vohwinkel G., Fritzsche B., Bokemeyer C., Wellbrock J., Fiedler W. (2021). Bone Marrow-Resident Vδ1 T Cells Co-express TIGIT with PD-1, TIM-3 or CD39 in AML and Myeloma. Front. Med..

[47] Peters C., Oberg H.-H., Kabelitz D., Wesch D. (2014). Phenotype and regulation of immunosuppressive Vδ2-expressing γδ T cells. Cell Mol. Life Sci..

