# Supplementary material for: Programmed Cell Death-1 Expression in T-Cell Subsets in Chickens Infected with Marek’s Disease Virus
Source: Pathogens. 2025 Apr 29;14(5):431. doi: 10.3390/pathogens14050431 (PMC12114408; doi:10.3390/pathogens14050431)
Supplement: Supplementary file 1 [file pathogens-14-00431-s001.zip › pathogens-3577136-supplementary.pptx]

## Slide 1
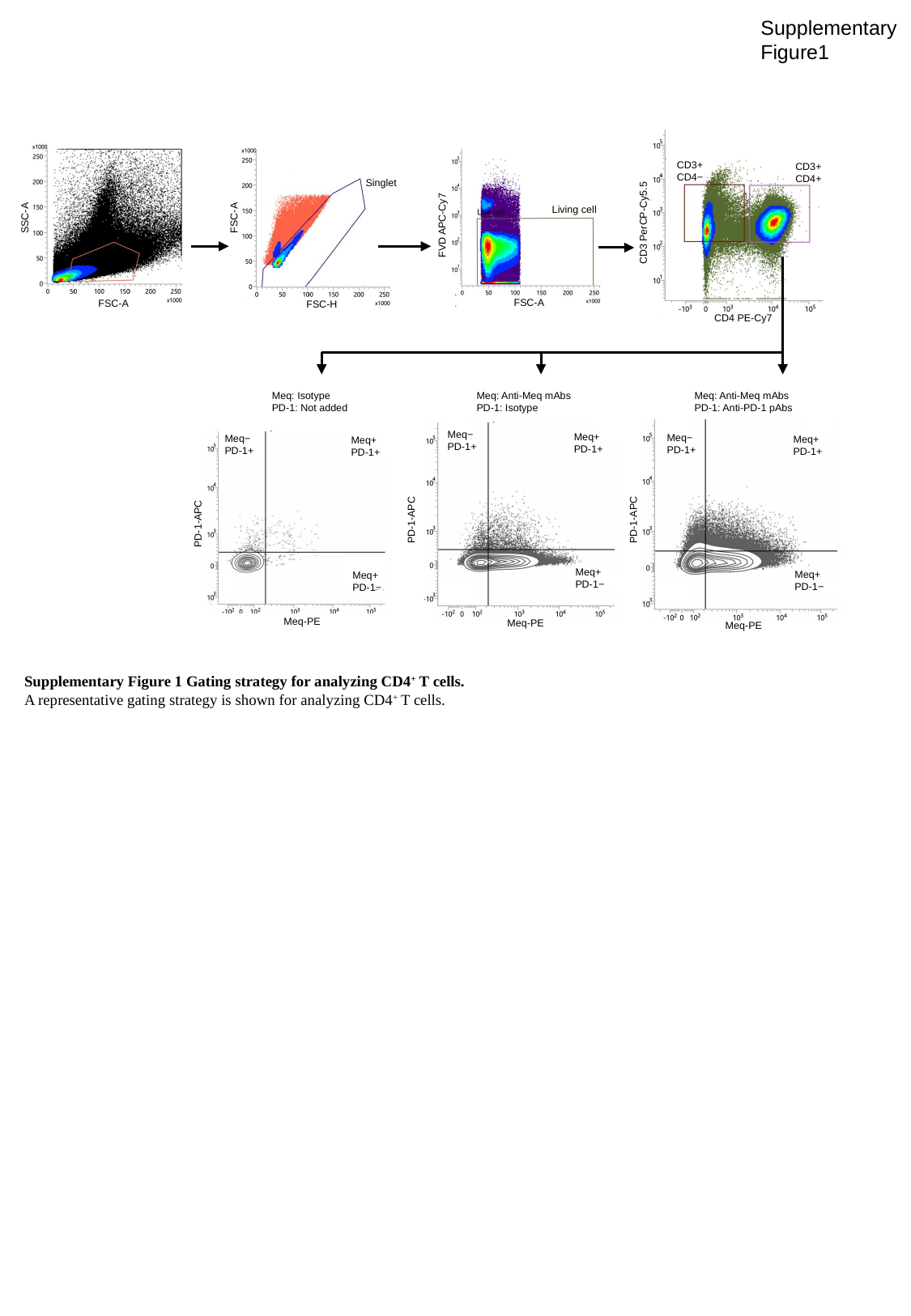

Supplementary Figure1
CD3+
CD4−
CD3+
CD4+
Singlet
Living cell
FSC-A
SSC-A
CD3 PerCP-Cy5.5
FVD APC-Cy7
FSC-A
FSC-A
FSC-H
CD4 PE-Cy7
Meq: Isotype
PD-1: Not added
Meq−
PD-1+
Meq+
PD-1+
PD-1-APC
Meq+
PD-1−
Meq-PE
Meq: Anti-Meq mAbs
PD-1: Isotype
Meq−
PD-1+
Meq+
PD-1+
PD-1-APC
Meq+
PD-1−
Meq-PE
Meq: Anti-Meq mAbs
PD-1: Anti-PD-1 pAbs
Meq−
PD-1+
Meq+
PD-1+
PD-1-APC
Meq+
PD-1−
Meq-PE
Supplementary Figure 1 Gating strategy for analyzing CD4+ T cells.
A representative gating strategy is shown for analyzing CD4+ T cells.

## Slide 2
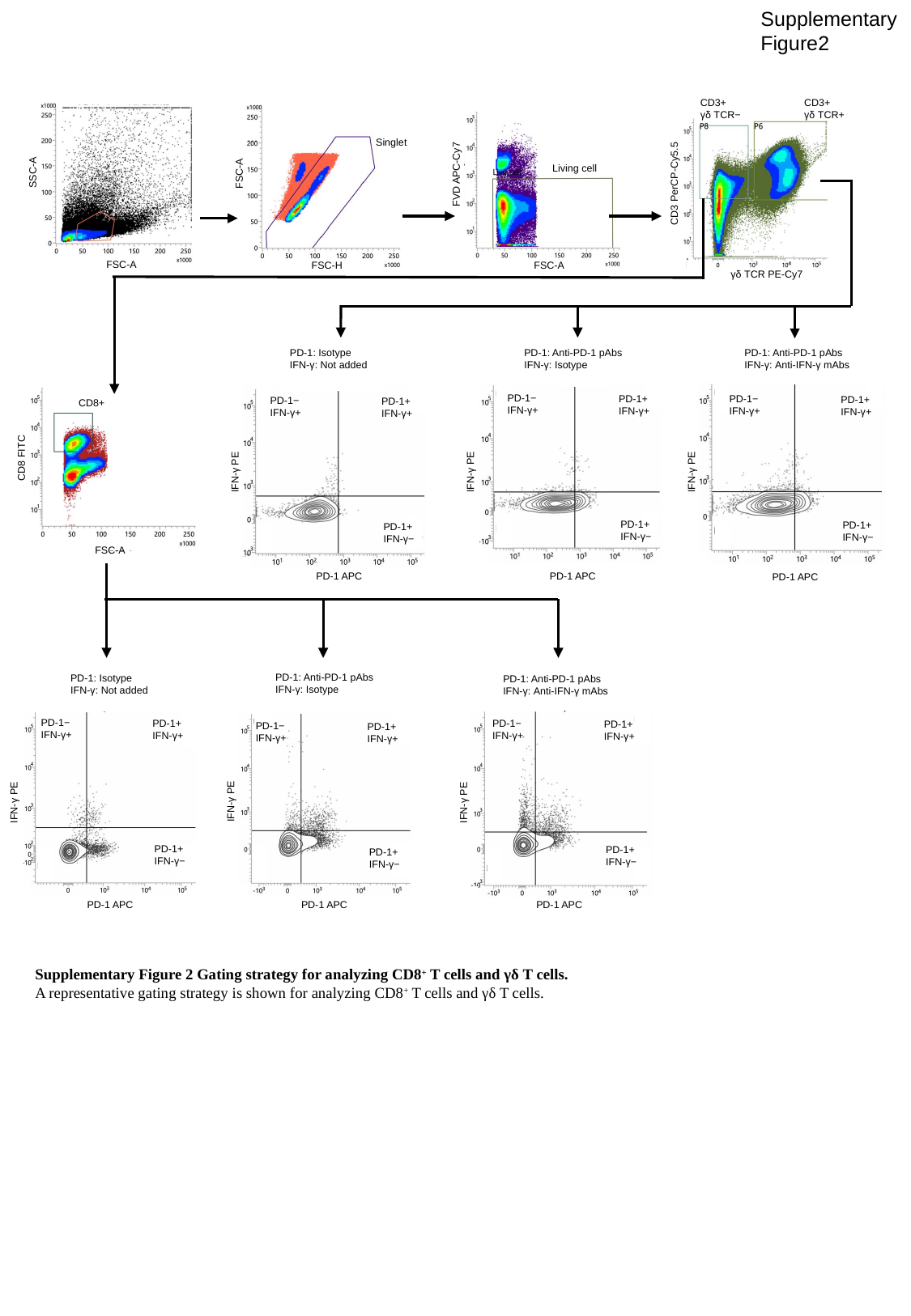

Supplementary Figure2
CD3+
γδ TCR+
CD3+
γδ TCR−
Singlet
Living cell
SSC-A
FSC-A
FVD APC-Cy7
CD3 PerCP-Cy5.5
FSC-A
FSC-A
FSC-H
 γδ TCR PE-Cy7
PD-1: Isotype
IFN-γ: Not added
PD-1: Anti-PD-1 pAbs
IFN-γ: Isotype
PD-1: Anti-PD-1 pAbs
IFN-γ: Anti-IFN-γ mAbs
PD-1−
IFN-γ+
PD-1−
IFN-γ+
PD-1+
IFN-γ+
PD-1+
IFN-γ+
PD-1−
IFN-γ+
PD-1+
IFN-γ+
CD8+
CD8 FITC
IFN-γ PE
IFN-γ PE
IFN-γ PE
PD-1+
IFN-γ−
PD-1+
IFN-γ−
PD-1+
IFN-γ−
FSC-A
PD-1 APC
PD-1 APC
PD-1 APC
PD-1: Anti-PD-1 pAbs
IFN-γ: Isotype
PD-1: Isotype
IFN-γ: Not added
PD-1: Anti-PD-1 pAbs
IFN-γ: Anti-IFN-γ mAbs
PD-1−
IFN-γ+
PD-1−
IFN-γ+
PD-1+
IFN-γ+
PD-1+
IFN-γ+
PD-1−
IFN-γ+
PD-1+
IFN-γ+
IFN-γ PE
IFN-γ PE
IFN-γ PE
PD-1+
IFN-γ−
PD-1+
IFN-γ−
PD-1+
IFN-γ−
PD-1 APC
PD-1 APC
PD-1 APC
Supplementary Figure 2 Gating strategy for analyzing CD8+ T cells and γδ T cells.
A representative gating strategy is shown for analyzing CD8+ T cells and γδ T cells.

## Slide 3
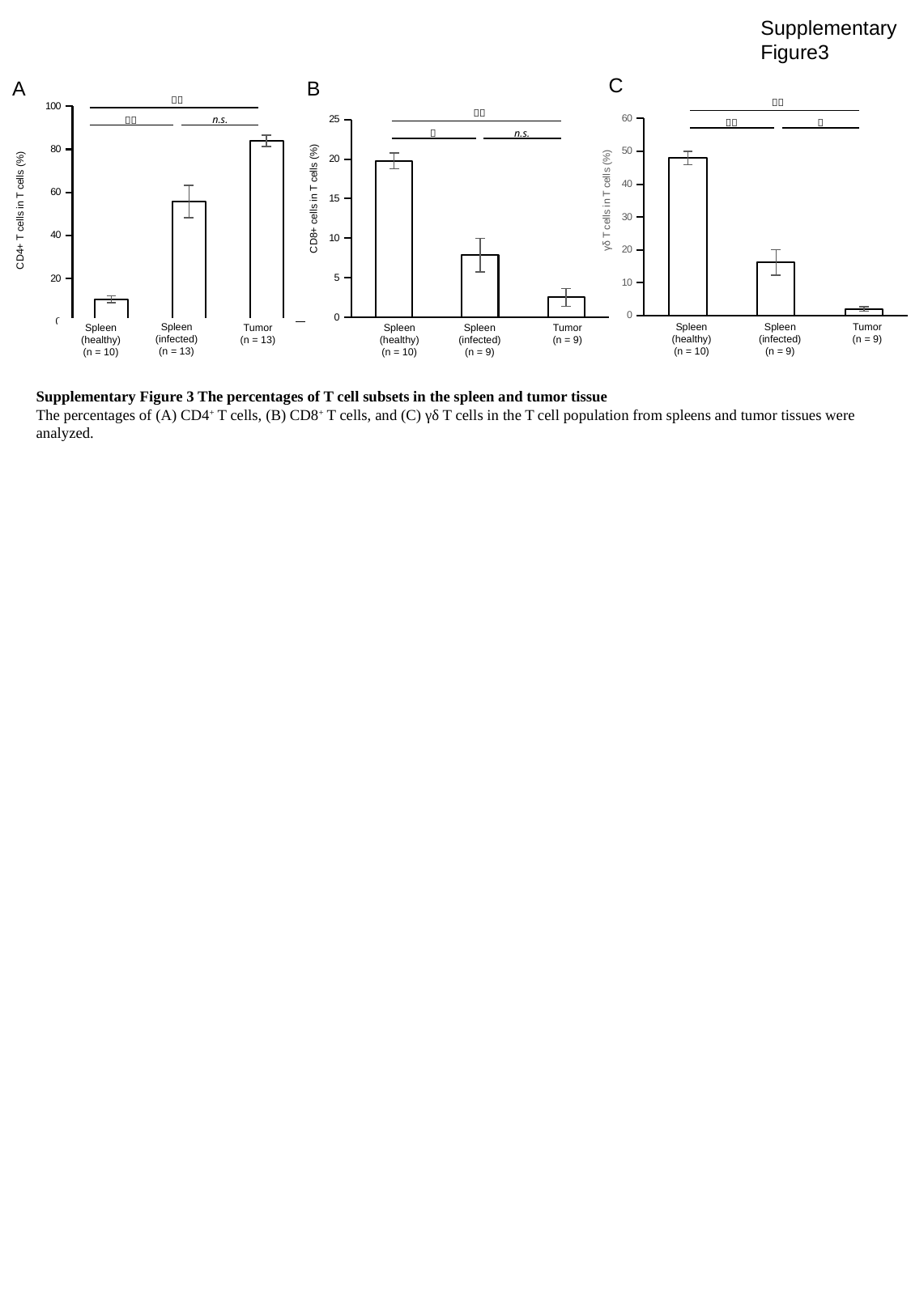

Supplementary Figure3
C
A
B
＊＊
＊＊
### Chart
| Category | |
|---|---|
| Spleen (healthy) | 10.411999999999997 |
| Spleen((infected) | 55.802 |
| Tumor | 83.92250000000001 |
### Chart
| Category | |
|---|---|
| Spleen (healthy) | 48.00224830432671 |
| Spleen (infected) | 16.144303310268533 |
| Tumor | 1.941899604445485 |
### Chart
| Category | |
|---|---|
| Spleen (healthy) | 19.78417242231306 |
| Spleen (infected) | 7.839454078437337 |
| Tumor | 2.522736067956102 |＊＊
n.s.
＊＊
＊
＊＊
n.s.
＊
Tumor
(n = 9)
Spleen
(infected)
(n = 13)
Spleen
(healthy)
(n = 10)
Spleen
(infected)
(n = 9)
Spleen
(healthy)
(n = 10)
Spleen
(infected)
(n = 9)
Tumor
(n = 9)
Spleen
(healthy)
(n = 10)
Tumor
(n = 13)
Supplementary Figure 3 The percentages of T cell subsets in the spleen and tumor tissue
The percentages of (A) CD4+ T cells, (B) CD8+ T cells, and (C) γδ T cells in the T cell population from spleens and tumor tissues were analyzed.

## Slide 4
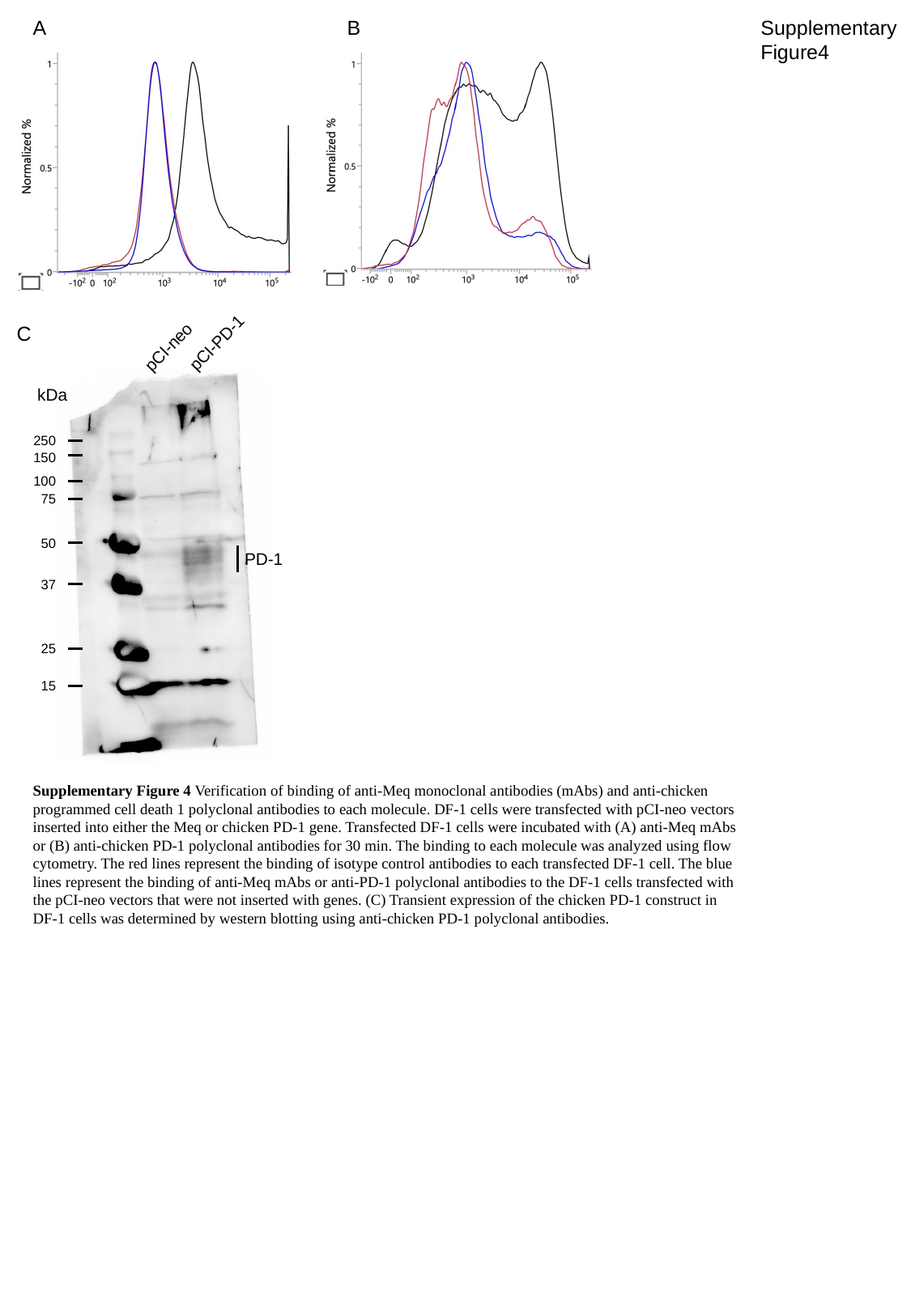

A
B
Supplementary Figure4
pCI-PD-1
pCI-neo
C
kDa
250
150
100
75
50
PD-1
37
25
15
Supplementary Figure 4 Verification of binding of anti-Meq monoclonal antibodies (mAbs) and anti-chicken programmed cell death 1 polyclonal antibodies to each molecule. DF-1 cells were transfected with pCI-neo vectors inserted into either the Meq or chicken PD-1 gene. Transfected DF-1 cells were incubated with (A) anti-Meq mAbs or (B) anti-chicken PD-1 polyclonal antibodies for 30 min. The binding to each molecule was analyzed using flow cytometry. The red lines represent the binding of isotype control antibodies to each transfected DF-1 cell. The blue lines represent the binding of anti-Meq mAbs or anti-PD-1 polyclonal antibodies to the DF-1 cells transfected with the pCI-neo vectors that were not inserted with genes. (C) Transient expression of the chicken PD-1 construct in DF-1 cells was determined by western blotting using anti-chicken PD-1 polyclonal antibodies.
